# Supplementary figures and images for: Computationally accelerated identification of P-glycoprotein inhibitors
Source: PLoS One. 2025 Aug 13;20(8):e0325121. doi: 10.1371/journal.pone.0325121 (PMC12349723; doi:10.1371/journal.pone.0325121)

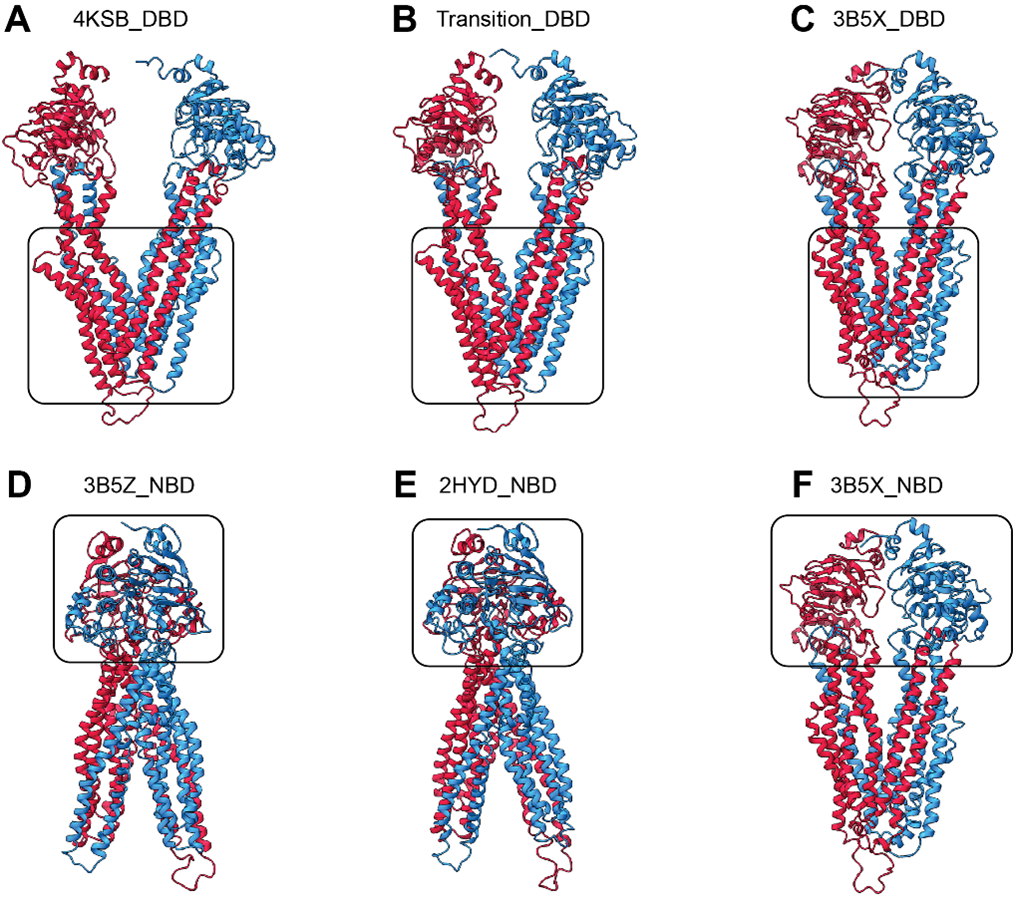

Supplement: S1 Fig — Panel 1: A. 4KSB_DBD, B. Transition_DBD, C. 3B5X_DBD, D. 3B5Z_NBD_1 and 3B5Z_NBD_2, E. 2HYD_NBD_1 and 2HYD_NBD_2, F. 3B5X_NBD. The NBD of the Transition structure in B was also sampled. NBD dock boxes for the 2HYD and 3B5Z structures were designed to sample each NBD individually. A-F are human P-gp structures. Boxes correspond to the region targeted using AutoDock Vina with an exhaustiveness of 128. (TIF) [file pone.0325121.s001.tif]

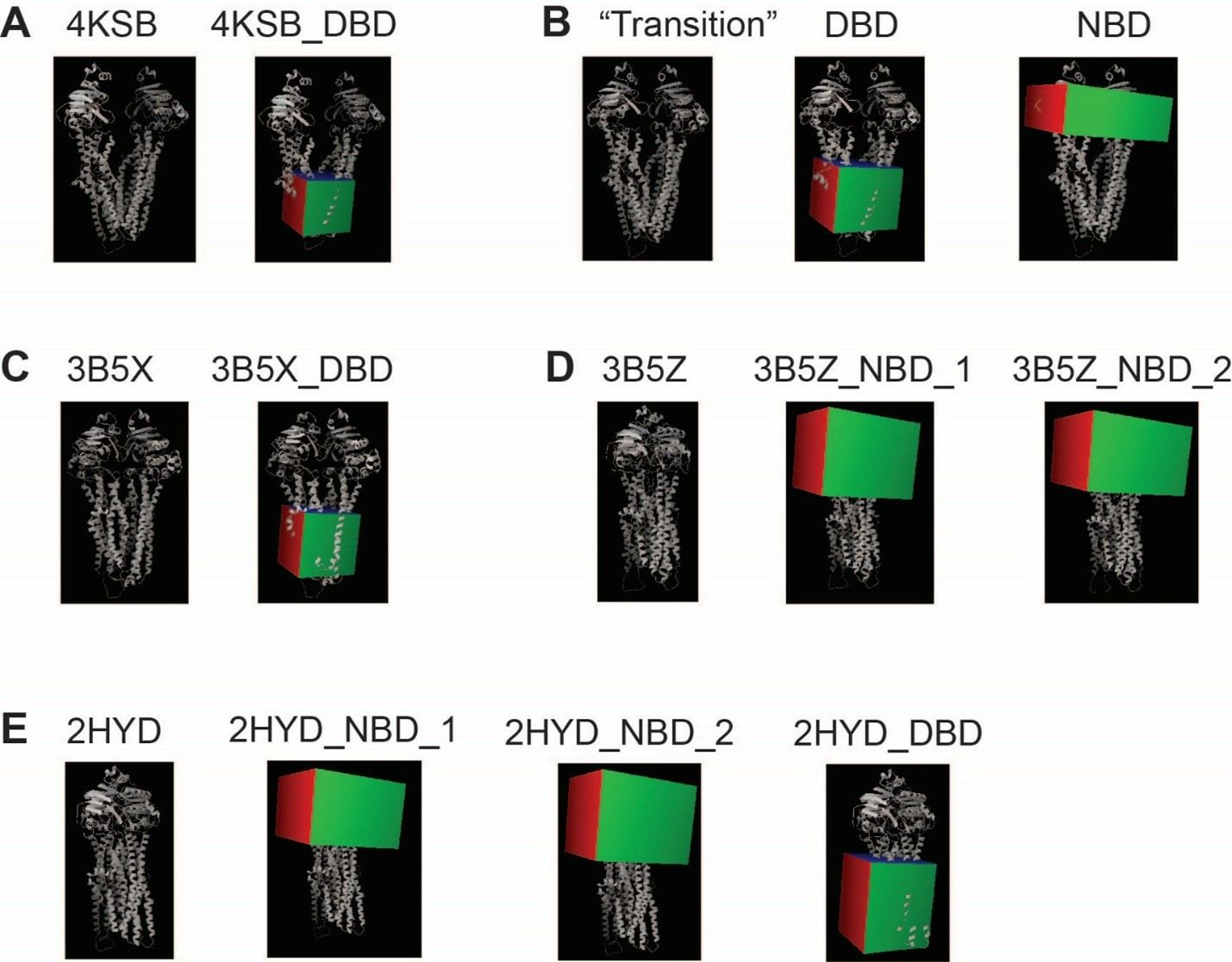

Supplement: S2 Fig — The conformations of the human P-gp model, and the corresponding dock boxes used, are shown in A – E. The first picture in each panel shows the receptor, with the PDB ID. The following pictures with red and green boxes show the docking boxes for the DBD or NBD of each receptor. Pictures generated using AutoDock. Note that the “Transition” structure is a conformation in-between 4KSB and 3B5X and is derived from TMD simulations performed for this study. (TIF) [file pone.0325121.s002.tif]

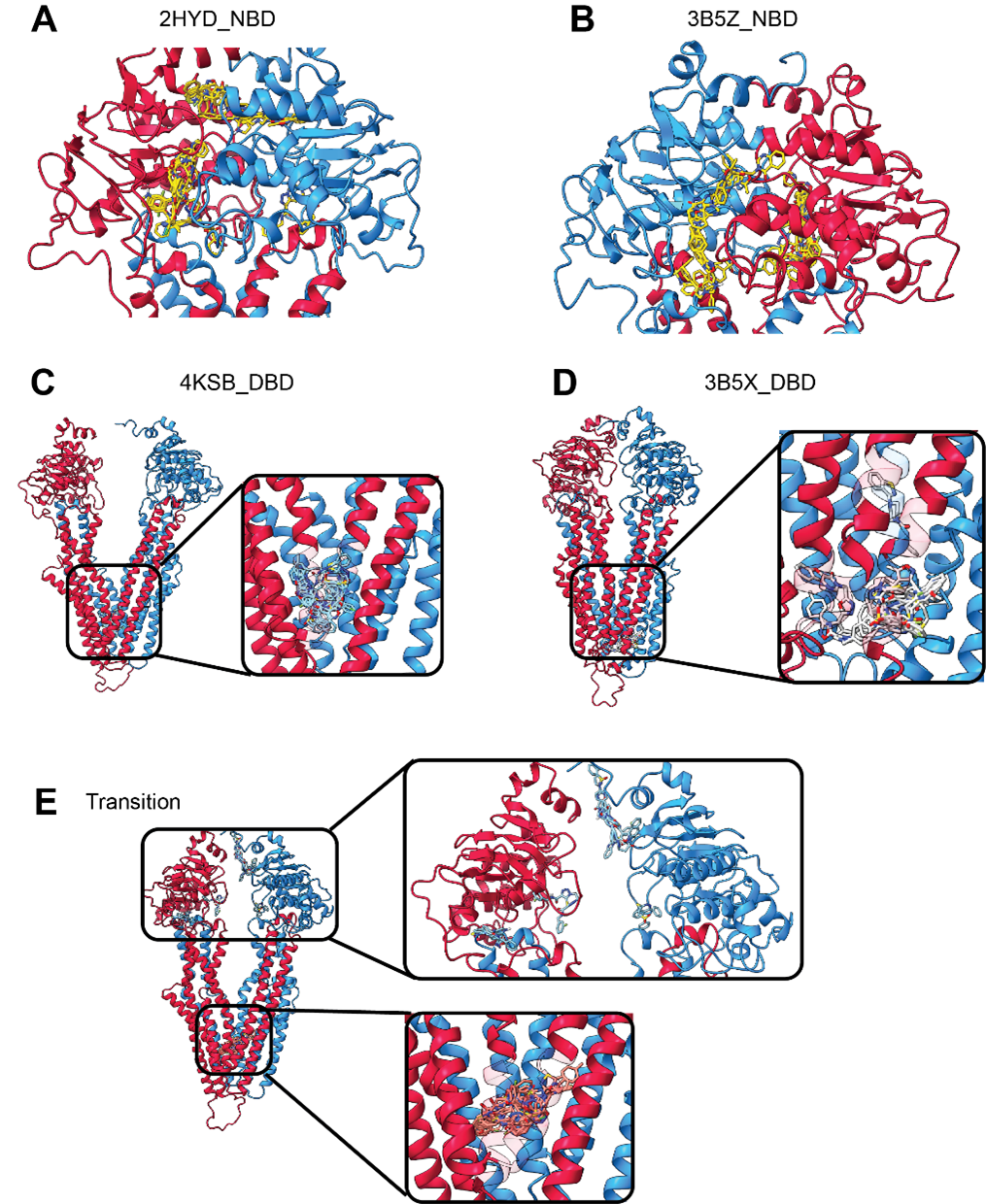

Supplement: S3 Fig — The docking positions of the top hits to the NBDs of A) 2HYD and B) 3B5Z were mostly located within the ATP-binding site, with the exception of a few positions above the ATP-binding site in 2HYD. The docking positions of top hits to the DBDs of C) 4KSB and D) 3B5X were near the middle or the bottom of the drug binding region, respectively. Ligands were docked to both the E, top box) NBDs and E, bottom box) DBDs of the “Transition” structure, which was generated from MD simulation trajectories that transitioned the protein from the 4KSB to the 3B5X conformation. It is notable that several hits were predicted to bind near the ATP-binding sites, even though, when the NBDs are disengaged, the catalytic configuration for ATP hydrolysis is incomplete. (TIF) [file pone.0325121.s003.tif]

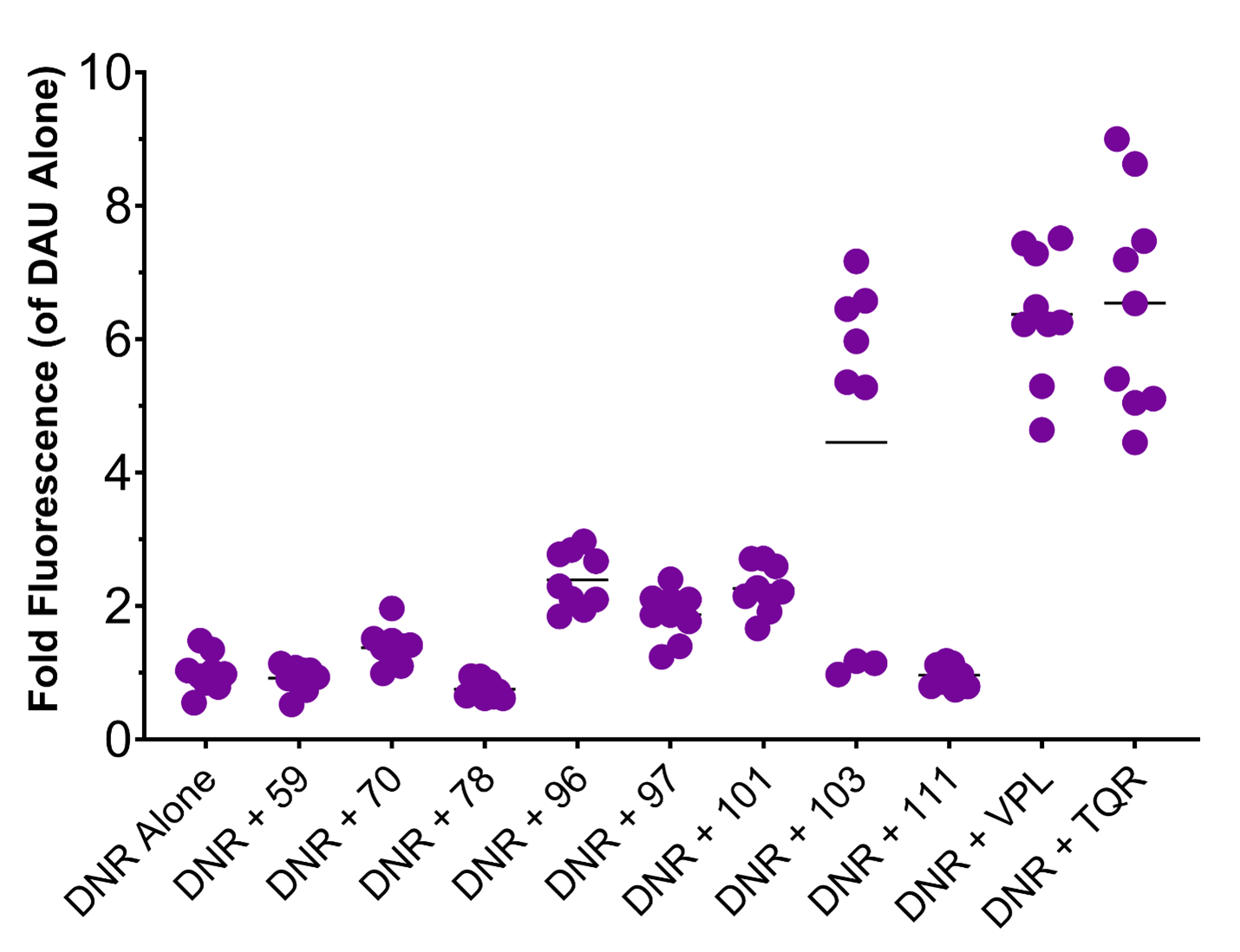

Supplement: S4 Fig — An alternative representation of the data in Fig 5. Fold change in fluorescence of the P-gp substrate Daunorubicin (DAU) in the presence or absence of experimental compounds or known P-gp modulators. Cells were treated with 10 µM compound in the presence or absence of 10 µM DAU. After washing and lysing the cells, the intracellular DAU fluorescence was measured and expressed as a fold change relative to the fluorescence of DU145-TXR cells treated with DAU alone. The P-gp inhibitors, VPL and TQR, were included as positive controls for P-gp inhibition, and compound 59 was included as a negative control for P-gp inhibition. Three samples per trial, three independent trials. Significance was determined using a Student’s T test of the mean by comparing fluorescence of DAU and compound to that of DAU alone; P > 0.05 = N.S., P < 0.05 = *, P < 0.01 = **, P < 0.001 = ***, P < 0.0001 = ****. Note that for compound 78, the significance denotes a significant decrease in fluorescence. (TIF) [file pone.0325121.s004.tif]
