# Supplementary material for: Computationally accelerated identification of P-glycoprotein inhibitors
Source: PLoS One. 2025 Aug 13;20(8):e0325121. doi: 10.1371/journal.pone.0325121 (PMC12349723; doi:10.1371/journal.pone.0325121)
Supplement: S1 Table — A non-exhaustive list of known P-gp inhibitors and their selected chemical properties [72,73]. Clinical trial outcomes were adapted from [1,11]. Compounds were evaluated using the SWISS-ADME server [60]. RO5: Rule of 5, MW: Molecular Weight, TPSA: Topological Polar Surface area, LogP: Average of 5 calculations of LogPoct/wat, LogS: Average of 3 calculations to determine aqueous solubility – Insoluble, Poorly soluble (Poor), Moderately Soluble (Moderate), Soluble. “Predicted P-gp Substrate” – predicted by SWISS-ADME to be a transport substrate of P-gp. “Known P-gp Substrate” – In addition to being P-gp inhibitors, these molecules are: known transport substrates of P-gp, known non-transport substrates of P-gp, unknown whether it is a transport substrate of P-gp [74–76]. If the molecule is designated in a specific generation of P-gp inhibitors – First Generation P-gp inhibitors, Second generation P-gp inhibitors, Third generation P-gp inhibitors. (DOCX) [file pone.0325121.s005.docx]

**S1 Table. Known P-gp Inhibitors and Predicted Chemical Properties**. A non-exhaustive list of known P-gp inhibitors and their selected chemical properties ^[64, 65]^. Clinical trial outcomes were adapted from ^[10, 11]^. Compounds were evaluated using the SWISS-ADME server ^[54]^. RO5: Rule of 5, MW: Molecular Weight, TPSA: Topological Polar Surface area, LogP: Average of 5 calculations of LogP_oct/wat_ , LogS: Average of 3 calculations to determine aqueous solubility – Insoluble, Poorly soluble (Poor), Moderately Soluble (Moderate), Soluble. “Predicted P-gp Substrate” - predicted by SWISS-ADME to be a transport substrate of P-gp. “Known P-gp Substrate” – In addition to being P-gp inhibitors, these molecules are: known transport substrates of P-gp, known non-transport substrates of P-gp, unknown whether it is a transport substrate of P-gp ^[66-68]^. If the molecule is designated in a specific generation of P-gp inhibitors - First Generation P-gp inhibitors, Second generation P-gp inhibitors, Third generation P-gp inhibitors.

| **Generation of P-gp Inhibitors**  Generation of P-gp Inhibitors |  | **Compound** | **MW (g/mol)** | **Clinical Trial**  **Result** | **# RO5**  **Violations** | **TPSA (Å^2^)** | **Log P*_oct/wat_*** | **LogS** | **Predicted P-gp Substrate** | | **P-gp Substrate?** |
| --- | --- | --- | --- | --- | --- | --- | --- | --- | --- | --- | --- |
|  | 1^st^ | Verapamil | 455 | Fail | 0 | 64 | 4.45 | Moderate | | Yes | Yes |
|  |  | Reserpine | 609 |  | 2 | 118 | 3.52 | Poor | | Yes | No |
|  |  | Cyclosporin A | 1203 | Fail | 2 | 279 | 2.38 | Poor | | Yes | Yes |
|  |  | Quinidine | 324 | Fail | 0 | 46 | 2.81 | Soluble | | No | Yes |
|  |  | Amiodarone | 645 | Fail | 2 | 43 | 6.49 | Poor | | Yes | Yes |
|  | 2^nd^ | Valspodar | 1215 | Fail | 2 | 276 | 2.26 | Poor | | Yes | No |
|  |  | Elacridar | 564 | Fail | 1 | 93 | 4.93 | Poor | | No | Yes |
|  |  | Dexverapamil | 455 | Fail | 0 | 64 | 4.45 | Moderate | | Yes | No |
|  | 3^rd^ | Tariquidar | 647 | Fail | 1 | 111 | 5.2 | Poor | | No | Yes |
|  |  | Zosuquidar | 528 | Fail | 1 | 49 | 4.64 | Poor | | No | Yes |
