# Supplementary material for: Computationally accelerated identification of P-glycoprotein inhibitors
Source: PLoS One. 2025 Aug 13;20(8):e0325121. doi: 10.1371/journal.pone.0325121 (PMC12349723; doi:10.1371/journal.pone.0325121)
Supplement: S2 Table — (DOCX) [file pone.0325121.s006.docx]

**S2 Table. Parameters of dock boxes used for docking screens.**

|  |  |  | Center coordinates | | | Size (Å) | | |
| --- | --- | --- | --- | --- | --- | --- | --- | --- |
| Receptor Name | PDB source | Search Area | X | Y | Z | X | Y | Z |
| 2HYD_DBD_1 | 2HYD | DBD | -7.7 | 0.0 | 3.2 | 50 | 50 | 60 |
| 2HYD_NBD_1 | 2HYD | NBD | -4.3 | 2.5 | 70.1 | 76 | 64 | 58 |
| 2HYD_NBD_2 | 2HYD | NBD | -3.9 | 1.7 | 70.1 | 78 | 76 | 50 |
| 3B5Z_NBD_1 | 3B5Z | NBD | -4.3 | 2.5 | 70.1 | 76 | 64 | 58 |
| 3B5Z_NBD_2 | 3B5Z | NBD | -3.9 | 1.7 | 70.1 | 78 | 76 | 50 |
| 3B5X_DBD | 3B5X | DBD | -0.9 | 1.7 | 2.6 | 44 | 40 | 44 |
| Transition_NBD |  | NBD | -2.5 | 3.8 | 61.4 | 78 | 76 | 30 |
| Transition_DBD |  | DBD | -0.9 | 1.7 | 2.6 | 50 | 46 | 44 |
| 4KSB_DBD | 4KSB | DBD | -1.4 | 0.8 | -2.8 | 40 | 40 | 40 |
