# Supplementary material for: Computationally accelerated identification of P-glycoprotein inhibitors
Source: PLoS One. 2025 Aug 13;20(8):e0325121. doi: 10.1371/journal.pone.0325121 (PMC12349723; doi:10.1371/journal.pone.0325121)
Supplement: S3 Table — Ratios are calculated as the estimated DBD affinity (KDDBD) divided by the estimated NBD affinity (KDNBD). Ratios are listed by compound, and then organized by receptor structure. Three DBD docking boxes and five NBD boxes were used (Supplemental Figure 1). Each NBD of the 2HYD and 3B5Z structures was given its own docking box, hence the labels “nbd_1” and “nbd_2”. Molecules with a high ratio of DBD/NBD affinities are the most desirable, as this indicates that the KDestimate to the DBDs is much larger (and thus lower affinity) than the KDestimate to the NBDs. (DOCX) [file pone.0325121.s007.docx]

**S3 Table. Ratio of estimated affinities (K_D_^estimate^) for the top hits.** Ratios are calculated as the estimated DBD affinity (K_D_^DBD^) divided by the estimated NBD affinity (K_D_^NBD^). Ratios are listed by compound, and then organized by receptor structure. Three DBD docking boxes and five NBD boxes were used (Supplemental Figure 1). Each NBD of the 2HYD and 3B5Z structures was given its own docking box, hence the labels “nbd_1” and “nbd_2”. Molecules with a high ratio of DBD/NBD affinities are the most desirable, as this indicates that the K_D_^estimate^ to the DBDs is much larger (and thus lower affinity) than the K_D_^estimate^ to the NBDs.

| SMU ID | 4KSB DBD / NBD | | | | |
| --- | --- | --- | --- | --- | --- |
|  | 2hyd_nbd_1 | 2hyd_nbd_2 | 3b5z_nbd_1 | 3b5z_nbd_2 | transition_nbd |
| 70 | 3.3 | 57.3 | 3.9 | 3.3 | 6.4 |
| 78 | 1.0 | 1.0 | 1.2 | 0.2 | 0.4 |
| 96 | 0.3 | 0.4 | 0.0 | 0.0 | 0.0 |
| 97 | 2.8 | 0.7 | 1.0 | 0.1 | 0.1 |
| 101 | 2.0 | 40.9 | 5.4 | 2.8 | 2.8 |
| 103 | -- | 12.6 | 3.9 | 0.4 | 1.7 |
| 111 | 0.7 | 0.5 | 0.7 | 0.1 | 0.7 |
| 122 | 14.9 | 7.6 | 17.6 | 3.9 | 2.3 |
| 124 | 7.6 | 57.3 | 1.7 | 5.4 | 1.2 |
| SMU ID | 3B5X DBD / NBD | | | | |
|  | 2hyd_nbd_1 | 2hyd_nbd_2 | 3b5z_nbd_1 | 3b5z_nbd_2 | transition_nbd |
| 70 | 6.4 | 112.6 | 7.6 | 6.4 | 12.6 |
| 78 | 3.9 | 3.9 | 4.6 | 0.8 | 1.4 |
| 96 | 3.9 | 6.4 | 0.2 | 0.1 | 0.1 |
| 97 | 6.4 | 1.7 | 2.3 | 0.3 | 0.2 |
| 101 | 2.3 | 48.4 | 6.4 | 3.3 | 3.3 |
| 103 | -- | 14.9 | 4.6 | 0.5 | 2.0 |
| 111 | 6.4 | 4.6 | 6.4 | 1.2 | 6.4 |
| 122 | 7.6 | 3.9 | 9.0 | 2.0 | 1.2 |
| 124 | 9.0 | 67.9 | 2.0 | 6.4 | 1.4 |
| SMU ID | Transition DBD / NBD | | | | |
|  | 2hyd_nbd_1 | 2hyd_nbd_2 | 3b5z_nbd_1 | 3b5z_nbd_2 | transition_nbd |
| 70 | 0.8 | 14.9 | 1.0 | 0.8 | 1.7 |
| 78 | 1.7 | 1.7 | 2.0 | 0.4 | 0.6 |
| 96 | 4.6 | 7.6 | 0.3 | 0.2 | 0.1 |
| 97 | 2.3 | 0.6 | 0.8 | 0.1 | 0.1 |
| 101 | 1.2 | 24.7 | 3.3 | 1.7 | 1.7 |
| 103 | -- | 1.4 | 0.4 | 0.0 | 0.2 |
| 111 | 0.8 | 0.6 | 0.8 | 0.2 | 0.8 |
| 122 | 1.4 | 0.7 | 1.7 | 0.4 | 0.2 |
| 124 | 4.6 | 7.6 | 0.3 | 0.2 | 0.1 |
