# Supplementary material for: Computationally accelerated identification of P-glycoprotein inhibitors
Source: PLoS One. 2025 Aug 13;20(8):e0325121. doi: 10.1371/journal.pone.0325121 (PMC12349723; doi:10.1371/journal.pone.0325121)
Supplement: S4 Table — DU145-TXR cells were incubated with 15 µM compound with or without 500nM PTX for 48 hours; survival was subsequently determined with the Resazurin viability assay [39]. Data represent the mean of two separate experiments performed in triplicate, and shows viability of cells treated with 15 µM compound alone, 15 µM compound and 500 nM PTX, and the difference between survivability measurements of each treatment. Percent re-sensitization is defined as the percent change in viability between cells treated with compound and PTX, versus cells treated with PTX alone. In some instances, the assay reported an increase in viability with compound + PTX, and no percent sensitization is reported. ‘Follow-up Testing’ compounds were re-assessed with MTT assays (Figure 1). Compounds 59 and 89 (*, ‘Follow-up Testing’) were used as negative controls in follow-up MTT assays to test for consistency of results, e.g., to confirm that molecules eliminated in Resazurin assays were justifiably eliminated from later screening with MTT assays. Tariquidar was included as a positive control for P-gp inhibition. (DOCX) [file pone.0325121.s008.docx]

**S4 Table**. **Pre-screening of compounds 56 – 98 against DU145-TXR cells with resazurin assays**. DU145-TXR cells were incubated with 15 µM compound with or without 500nM PTX for 48 hrs; survival was subsequently determined with the Resazurin viability assay ^[37]^. Data represent the mean of two separate experiments performed in triplicate, and shows viability of cells treated with 15 µM compound alone, 15 µM compound and 500 nM PTX, and the difference between survivability measurements of each treatment. Percent re-sensitization is defined as the percent change in viability between cells treated with compound and PTX, versus cells treated with PTX alone. In some instances, the assay reported an increase in viability with compound + PTX, and no percent sensitization is reported. ‘Follow-up Testing’ compounds were re-assessed with MTT assays (Figure 1). Compounds 59 and 89 (*, ‘Follow-up Testing’) were used as negative controls in follow-up MTT assays to test for consistency of results, e.g. to confirm that molecules eliminated in Resazurin assays were justifiably eliminated from later screening with MTT assays. Tariquidar was included as a positive control for P-gp inhibition.

|  | 15 µM compound alone | 15 µM compound + 500 nM PTX | Change in viability | Percent (%) sensitization | Followup testing? |
| --- | --- | --- | --- | --- | --- |
| 56 | 96.3 | 96.9 | +0.6 |  |  |
| 57 | 70.3 | 64.8 | 5.5 | 7.8 |  |
| 58 | 99.9 | 97.8 | 2.1 | 2.1 |  |
| 59 | 101.1 | 100.8 | 0.3 | 0.3 | * |
| 60 | 88.0 | 47.0 | 41 | 46.6 | Yes |
| 61 | 98.8 | 53.1 | 45.7 | 46.3 | Yes |
| 62 | 100.6 | 99.2 | 1.4 | 1.4 |  |
| 63 | 97.6 | 100.8 | +3.2 |  |  |
| 64 | 94.7 | 98.2 | +3.5 |  |  |
| 65 | 94.8 | 99.1 | +4.3 |  |  |
| 66 | 98.3 | 46.0 | 52.3 | 53.2 | Yes |
| 67 | 89.8 | 92.6 | +2.8 |  |  |
| 68 | 86.6 | 54.4 | 32.2 | 37.2 | Yes |
| 69 | 106.3 | 101.7 | 4.6 | 4.3 |  |
| 70 | 99.2 | 61.6 | 37.6 | 37.9 | Yes |
| 71 | 93.0 | 38.2 | 54.8 | 58.9 | Yes |
| 72 | 95.3 | 75.2 | 20.1 | 21.1 |  |
| 73 | 101.2 | 97.7 | 3.5 | 3.5 |  |
| 74 | 73.3 | 45.9 | 27.4 | 37.4 | Yes |
| 75 | 99.8 | 97.6 | 2.2 | 2.2 |  |
| 76 | 103.5 | 100.5 | 3.0 | 2.9 |  |
| 77 | 95.7 | 93.5 | 2.2 | 2.3 |  |
| 78 | 95.2 | 14.5 | 80.7 | 84.8 | Yes |
| 79 | 89.9 | 51.2 | 38.7 | 43.0 | Yes |
| 80 | 95.4 | 95.1 | 0.3 | 0.3 |  |
| 81 | 77.4 | 74.8 | 2.6 | 3.4 |  |
| 82 | 97.7 | 96.7 | 1.0 | 1.0 |  |
| 83 | 98.0 | 79.0 | 19.0 | 19.4 |  |
| 84 | 101.9 | 105.6 | +3.7 |  |  |
| 85 | 99.5 | 100.9 | +1.4 |  |  |
| 86 | 97.3 | 96.8 | 0.5 | 0.5 |  |
| 87 | 53.1 | 53.4 | +0.3 |  |  |
| 88 | 94.1 | 90.2 | 3.9 | 4.1 |  |
| 89 | 93.7 | 92.9 | 0.8 | 0.9 | * |
| 90 | 97.4 | 95.5 | 1.9 | 2.0 |  |
| 91 | 99.6 | 94.0 | 5.6 | 5.6 |  |
| 92 | 99.9 | 98.6 | 1.3 | 1.3 |  |
| 93 | 100.5 | 97.7 | 2.8 | 2.8 |  |
| 94 | 98.8 | 96.3 | 2.5 | 2.5 |  |
| 95 | 95.6 | 77.8 | 17.8 | 18.6 |  |
| 96 | 89.4 | 16.1 | 73.3 | 82.0 | Yes |
| 97 | 102.2 | 63.6 | 38.6 | 37.8 | Yes |
| 98 | 97.4 | 80.1 | 17.3 | 17.8 |  |
| TQR | 75.2 | 9.1 | 66.2 | 87.9 |  |
