# Supplementary material for: Computationally accelerated identification of P-glycoprotein inhibitors
Source: PLoS One. 2025 Aug 13;20(8):e0325121. doi: 10.1371/journal.pone.0325121 (PMC12349723; doi:10.1371/journal.pone.0325121)
Supplement: S5 Table — Samples were prepared in triplicate and two or three independent trials were performed; data represent the mean ± one standard deviation (std. dev). In contrast to the methods used in [9], these LC-MS/MS trials measured the normalized ratio of analyte (i.e., compound) to the internal standard (i.e., unique performance of mass spectrometer on date of analysis); thus the data represent the relative quantification of compound per sample and explain the variability between independent trials (see Methods). (DOCX) [file pone.0325121.s009.docx]

**S5 Table. Normalized ratio of compound to internal standard using LC-MS/MS**. DU145-TXR cells were exposed to 5 µM compound with or without 500 nM tariquidar (TQR) as described in ^[8]^. Samples were prepared in triplicate and two or three independent trials were performed; data represent the mean ± one standard deviation (std. dev). In contrast to the methods used in ^[8]^, these LC-MS/MS trials measured the normalized ratio of analyte (i.e. compound) to the internal standard (i.e. unique performance of mass spectrometer on date of analysis); thus the data represent the relative quantification of compound per sample and explain the variability between independent trials (see Methods).

|  | **- TQR** | **+ TQR** | **P** | **- TQR** | **+ TQR** | **P** | **- TQR** | **+ TQR** | **P** |
| --- | --- | --- | --- | --- | --- | --- | --- | --- | --- |
| **DAU** | 0.014 ± 0.001 | 0.216 ± 0.002 | < 1x10^-6^ | 0.017 ± 0.001 | 0.232 ± 0.016 | 2x10^-5^ |  |  |  |
| **60** | 0.231 ± 0.165 | 0.282 ± 0.010 | 0.6 | 0.250 ± 0.003 | 0.250 ± 0.003 | > 1 |  |  |  |
| **61** | 0.023 ± 0.002 | 0.076 ± 0.001 | 2 x 10^-6^ | 0.016 ± 0.006 | 0.027 ± 0.011 | 0.2 | 0.021 ± 0.004 | 0.126 ± 0.010 | 0.0001 |
| **66** | 0.747 ± 0.096 | 0.833 ± 0.124 | 0.4 | 0.040 ± 0.001 | 0.036 ± 0.005 | 0.2 | 0.170 ± 0.039 | 0.140 ± 0.029 | 0.3 |
| **70** | 0.93 ± 0.15 | 0.93 ± 0.07 | >1 | 0.56 ± 0.11 | 0.55 ± 0.15 | 0.9 |  |  |  |
| **71** | 0.085 ± 0.013 | 0.067 ± 0.005 | 0.09 | 0.071 ± 0.009 | 0.091 ± 0.043 | 0.5 |  |  |  |
| **74** | 0.014 ± 0.000 | 0.014 ± 0.001 | > 1 | 0.028 ± 0.009 | 0.030 ± 0.005 | 0.8 | 11.51 ± 1.305 | 13.57 ± 0.248 | 0.05 |
| **78** | 0.001 ± 0.000 | 0.001 ± 0.000 | -- | 0.001 ± 0.000 | 0.001 ± 0.000 | -- |  |  |  |
| **96** | 0.087 ± 0.009 | 0.092 ± 0.036 | 0.8 | 0.121 ± 0.005 | 0.163 ± 0.032 | 0.09 |  |  |  |
| **97** | 0.07 ± 0.02 | 0.08 ± 0.03 | 0.7 | 0.10 ± 0.02 | 0.10 ± 0.02 | > 1 |  |  |  |
| **101** | 11.86 ± 1.16 | 12.03 ± 2.45 | 0.9 | 21.17 ± 3.59 | 22.88 ± 2.92 | 0.6 |  |  |  |
| **103** | 5.13 ± 0.6 | 5.61 ± 1.34 | 0.6 | 2.63 ± 0.52 | 3.75 ± 0.34 | 0.04 | 3.21 ± 0.23 | 3.73 ± 0.15 | 0.03 |
| **111** | 0.012 ± 0.002 | 0.013 ± 0.004 | 0.7 | 0.011 ± 0.002 | 0.013 ± 0.001 | 0.2 |  |  |  |
| **122** | 0.049 ± 0.005 | 0.146 ± 0.018 | 0.0008 | 0.058 ± 0.018 | 0.209 ± 0.014 | 0.0003 |  |  |  |
| **124** | 0.006 ± 0.001 | 0.061 ± 0.011 | 0.001 | 0.007 ± 0.002 | 0.055 ± 0.009 | 0.0008 |  |  |  |
