# Supplementary material for: Computationally accelerated identification of P-glycoprotein inhibitors
Source: PLoS One. 2025 Aug 13;20(8):e0325121. doi: 10.1371/journal.pone.0325121 (PMC12349723; doi:10.1371/journal.pone.0325121)
Supplement: S6 Table — Here we show the fold fluorescence of the P-gp substrate Daunorubicin (DAU) in the presence or absence of experimental compounds or known P-gp modulators. Cells were treated with 10 µM compound in the presence or absence of 10 µM DAU, after which cells were washed and lysed; the resultant DAU fluorescence was measured on the Cytation 5 (excitation/emission 488 nm/ 575 nm). The resultant DAU fluorescence is expressed as a fold change relative to the fluorescence of DU145-TXR cells treated with DAU alone. The P-gp inhibitors VPL and TQR were included as positive controls for P-gp inhibition, and compound 59 was included as a negative control for P-gp inhibition. Three samples per trial, three independent trials. Statistical significance determined using GraphPad Prism, Student’s T test of the mean, by comparing the mean fluorescence of DAU + compound to that of DAU alone. Significance was determined using a Student’s T test of the mean; P > 0.05 = N.S., P < 0.05 = *, P < 0.01 = **, P < 0.001 = ***, P < 0.0001 = ****. (DOCX) [file pone.0325121.s010.docx]

**S6 Table. Fold Accumulation of Daunorubicin fluorescence in DU145-TXR cells.** Here we show the fold fluorescence of the P-gp substrate Daunorubicin (DAU) in the presence or absence of experimental compounds or known P-gp modulators. Cells were treated with 10 µM compound in the presence or absence of 10 µM DAU, after which cells were washed and lysed; the resultant DAU fluorescence was measured on the Cytation 5 (excitation/emission 488 nm / 575 nm). The resultant DAU fluorescence is expressed as a fold change relative to the fluorescence of DU145-TXR cells treated with DAU alone. The P-gp inhibitors VPL and TQR were included as positive controls for P-gp inhibition, and compound 59 was included as a negative control for P-gp inhibition. Three samples per trial, three independent trials. Statistical significance determined using GraphPad Prism, Student’s T test of the mean, by comparing the mean fluorescence of DAU + compound to that of DAU alone. Significance was determined using a Student’s T test of the mean; P > 0.05 = N.S., P < 0.05 = *, P < 0.01 = **, P < 0.001 = ***, P < 0.0001 = ****.

|  | **Fold Δ** | **P Value** | **Significance** |
| --- | --- | --- | --- |
| **DAU Alone** | 1.0 ± 0.3 |  |  |
| **DAU + 59** | 0.9 ± 0.2 | P = 0.48 | N.S. |
| **DAU + 70** | 1.4 ± 0.3 | P = 0.01 | * |
| **DAU + 78** | 0.75 ± 0.1 | P = 0.03 | * |
| **DAU + 96** | 2.4 ± 0.4 | P < 0.0001 | **** |
| **DAU + 97** | 1.9 ± 0.4 | P < 0.0001 | **** |
| **DAU + 101** | 2.3 ± 0.4 | P < 0.0001 | **** |
| **DAU + 103** | 4.5 ± 2.6 | P = 0.001 | ** |
| **DAU + 111** | 1.0 ± 0.2 | P = 0.75 | N.S. |
| **DAU + VPL** | 6.3 ± 1.0 | P < 0.0001 | **** |
| **DAU + TQR** | 6.5 ± 1.6 | P < 0.0001 | **** |
