# Supplementary material for: Computationally accelerated identification of P-glycoprotein inhibitors
Source: PLoS One. 2025 Aug 13;20(8):e0325121. doi: 10.1371/journal.pone.0325121 (PMC12349723; doi:10.1371/journal.pone.0325121)
Supplement: S7 Table — ZINC IDs are shown if available. Molecular structures are translated into SMILES, and the corresponding InChl keys are provided as well. (DOCX) [file pone.0325121.s011.docx]

**S7 Table. Alternative identifiers for molecules identified in this study.** ZINC IDs are shown if available. Molecular structures are translated into SMILES, and the corresponding InChl keys are provided as well.

| **#** | **SMILES** | **InChl Key** | **ZINC** |
| --- | --- | --- | --- |
| **59** | Cn1cnnc1SCc1c(nnn1-c1nonc1N)C(O)=O | HRULNCRRUBFEHC-UHFFFAOYSA-N | 130193 |
| **60** | Nc1nonc1-n1nnc(C(=O)N\N=C\c2c3ccccc3cc3ccccc23)c1COc1ccc(F)cc1 | IUYYNPMYIXKERT-AMVVHIIESA-N | 2394477 |
| **61** | CCN1CCN(Cc2c(nnn2-c2nonc2N)C(=O)N\N=C2\C(=O)N(C)c3ccccc23)CC1 | AIPNQMGXBPYYIH-XQNSMLJCSA-N | 22874636 |
| **66** | O=C(N1CCN(CC1)S(=O)(=O)c1cccc2nonc12)c1cc2ccccc2[nH]1 | ANFMLYJTRAARTN-UHFFFAOYSA-N | 13727116 |
| **70** | O=C(N1CCN(CC1)c1nsc2ccccc12)c1nc(no1)-c1ccc2cc[nH]c2c1 | WFCVBKDGPGPBHN-UHFFFAOYSA-N | 12389647 |
| **71** | COc1ccc(OC)c(NC(=O)c2nnn(c2C)-c2ccc3noc(-c4ccccc4)c3c2)c1 | XLBUSWFGBQBUHE-UHFFFAOYSA-N | 12274469 |
| **78** | Cc1onc(c1-c1nnc(Cn2cnc3c4ccccc4oc3c2=O)o1)-c1ccccc1 | RZONHOSTPSUOJP-UHFFFAOYSA-N | 31744412 |
| **79** | Cc1cccc(c1)C1Cc2c(cnn2-c2nc3ccccc3[nH]2)C(=O)C1 | JBULLGGIBFROGV-UHFFFAOYSA-N | 9421694,94169 |
| **89** | NS(=O)(=O)c1cccc(NC(=O)CSc2nnnn2C2CC2)c1 | BFXWBYBENJOJND-UHFFFAOYSA-N | 12808366 |
| **96** | O=S(=O)(CCSc1nnc(-c2ccccc2)c2ccccc12)c1ccccc1 | KHUMXFJGOTYFBR-UHFFFAOYSA-N | 84559953 |
| **97** | OC(Cn1c(CS(=O)(=O)c2ccccc2)nc2ccccc12)c1ccc(Cl)cc1 | VJMMFJLYEGJEAG-UHFFFAOYSA-N | 12577459 |
| **101** | COc1cccc(c1)N1CCN(CC1)c1ccc2nnc(CCC(=O)Nc3ccc(F)c(F)c3)n2n1 | AWFWASCTYWHYJR-UHFFFAOYSA-N | 9118852 |
| **103** | Cc1ccc(cc1)-c1noc(n1)-c1cn(Cc2ccc(NS(=O)(=O)c3ccccc3F)cc2)cn1 | LZOCKGYCAZNWJT-UHFFFAOYSA-N | 32974765 |
| **111** | Cc1onc(c1-c1nnc(Cn2cnc3ccccc3c2=O)o1)-c1ccccc1 | BTXVFIOVOXQJSY-UHFFFAOYSA-N | 12922226 |
| **122** | Fc1cccc(CNC(=O)c2nnn3CCn4nnc(C(=O)NCc5cccc(F)c5)c4Sc23)c1 | WZQVWCJCOLIHHH-UHFFFAOYSA-N | 36489963 |
| **124** | O=C(N1CCN(CC1)c1nsc2ccccc12)c1nc(no1)-c1ccccn1 | NZHVFOMJJPILIA-UHFFFAOYSA-N | 12408039 |
